# Critical analysis of macular hole repair techniques: a comprehensive systematic review and meta-analysis comparing internal limiting membrane flap and internal limiting membrane peeling for any size of macular hole

**DOI:** 10.1186/s12886-025-04011-0

**Published:** 2025-04-07

**Authors:** Syed Muhammad Muneeb Akhtar, Syed Zia Saleem, Syed Ali Asad Rizvi, Areeba Fareed, Munazza Mumtaz, Shiza Saleem, Anusha Bai, Afsana Ansari Shaik, Robert Kirchoff, Muhammad Sohaib Asghar

**Affiliations:** 1 Department of Medicine, Dow University of Health Sciences, Karachi, Pakistan; 2 Department of Medicine, Karachi Medical and Dental College, Karachi, Pakistan; 3 Dr. Ruth K. M. Pfau Civil Hospital, Karachi, Pakistan; 4 Department of Medicine, Hamdard College of Medicine & Dentistry, Karachi, Pakistan; 5 Department of Medicine, Liaquat National Hospital & Medical College, Karachi, Pakistan; 6 Mayo Clinic, Rochester, MN USA; 7 Department of Internal Medicine, Mayo Clinic, Phoenix, AZ USA; 8 AdventHealth, Sebring, FL USA

**Keywords:** Inverted internal limiting membrane flap technique, Internal limiting membrane peeling, Macular hole, Meta-analysis

## Abstract

**Background:**

Macular holes (MHs) are a leading cause of visual impairment and blindness worldwide defined as a partial or full thickness anatomical defect in the fovea of the retina. ILM peeling is an effective surgical procedure to repair the defect. However, this approach lacks efficacy for larger macular holes. ILM flap is a novel technique with demonstrated efficacy for repair of larger defects.

**Objective:**

This systematic review and meta-analysis aims to compare the effectiveness of these two techniques in treating any size and type of MH.

**Methods:**

A comprehensive literature search was conducted in the PubMed, Medline, and Cochrane Library databases to identify the relevant articles. The primary outcome measures were MH closure rate and postoperative VA. The statistical power was ensured by performing heterogeneity, publication bias, sensitivity analysis, and subgroup analysis. Pooled odds ratios (ORs), mean differences (MD), and 95% confidence intervals (CIs) were calculated. All statistical analyses were performed using R Statistical Software and meta package v4.17-0.

**Results:**

A total of thirty-two studies, comprising nine RCTs and twenty three retrospective studies were included in this meta-analysis, which involved 1220 eyes in the ILM flap group and 1277 eyes in the ILM peeling group. The overall MH closure rate significantly favored ILM flap technique (OR = 2.47, CI = 1.58 to 3.87; *P* < 0.001; I²= 30%). The overall pooled result for postoperative VA, no significant difference was observed between the two surgical methods. However, it favored ILM flap technique on subgroup analysis based on study type and MH size specifically in the RCTS with macular hole size > 400 μm (MD = -0.13, 95% CI = -0.17 to -0.08, *p* < 0.01; I^2^ = 13%), as well as on subgrouping based on follow-up duration (MD = -0.11, 95% CI = -0.14 to -0.08, *p* < 0.01; I^2^ = 25%).

**Conclusion:**

ILM flap technique resulted in significantly better closure rate with all sizes of MHs, coupled with improved visual acuity in larger MHs and with follow-up duration.

**Supplementary Information:**

The online version contains supplementary material available at 10.1186/s12886-025-04011-0.

## Introduction

A macular hole (MH) is characterized as a structural defect, either partial or full thickness, within the foveolar region of the neurosensory retina. This defect is identified by the disruption of the retinal layers extending from the internal limiting membrane to the photoreceptor layer. It leads to significant impairment in central vision and is a prevalent cause of vision deterioration among elderly individuals. Common symptoms at presentation include metamorphopsia and visual deprivation, primarily due to the involvement of the central fovea. However, these symptoms can be ameliorated through successful surgical closure [1, 2].

The potential contribution of anteroposterior vitreous traction forces in the development of MH has long been a subject of inquiry. Subsequently, this theory gained support through biomicroscopic examinations, underscoring its significance [3–5]. Prior to the introduction of vitrectomy by Kelly and Wendel in 1991 [6], macular holes were considered incurable.

Currently, Internal limiting membrane (ILM) peeling is widely acknowledged as the most effective surgical approach for treating MH, boasting a success rate exceeding 90% in accordance with prior studies [7–11]. However, this success rate is not consistently high for larger macular holes exceeding 400 μm and has been reported to drop to as low as 60–70% [12, 13]. In 2010, a novel technique was introduced by Michalewska et al. [14], involving the use of an ILM flap to cover large idiopathic macular holes. In this method, the ILM is not entirely removed but is instead left attached to the edges of the macular hole and inverted to cover it. This approach significantly increased the success rate for the closure of larger macular holes to approximately 98%. Notably, numerous studies have shown that the ILM flap technique yields superior anatomical and visual outcomes when compared to the conventional ILM peeling approach, both for larger [13, 15–21] and smaller macular holes [22]. Nevertheless, some studies have suggested that the results are statistically similar [23–27], or even favor ILM peeling [28–30].

To the best of our knowledge, a similar meta-analysis, which exclusively focused on macular holes > 400 μm and compared the anatomical and visual outcomes of these two techniques, was published in 2021 [31]. However, it only included randomized clinical trials (RCTs), and since then a number of both original and retrospective studies have been published. Consequently, with the aim of augmenting the statistical robustness of preceding findings, we performed a comprehensive double-arm meta-analysis to assess the anatomical and visual function effectiveness of vitrectomy with the ILM flap technique for any size or type of MHs (both > 400 μm and < 400 μm) without retinal detachment, including both RCTs and retrospective studies.

## Methods

### Data sources and search

A comprehensive literature search was conducted in the PubMed, Medline, and Cochrane Library databases before November 2023 to identify relevant studies. The following medical subject heading terms and keywords were used for the database searches:

“ilm“[All Fields] AND (“peeled“[All Fields] OR “peeling“[All Fields] OR “peelings“[All Fields] OR “peels“[All Fields]) AND (“retinal perforations“[MeSH Terms] OR (“retinal“[All Fields] AND “perforations“[All Fields]) OR “retinal perforations“[All Fields] OR (“macular“[All Fields] AND “hole“[All Fields]) OR “macular hole“[All Fields]) AND “ilm“[All Fields] AND (“surgical flaps“[MeSH Terms] OR (“surgical“[All Fields] AND “flaps“[All Fields]) OR “surgical flaps“[All Fields] OR “flap“[All Fields]).

We applied no language restrictions, non-English studies were translated for interpretation using Google translate service. The relevant literature’s references were carefully checked for potential eligible studies. Disagreements were resolved through consensus and, when necessary, arbitration by a third researcher (SAAR).

### Inclusion and exclusion criteria

The inclusion criteria for eligibility were as follows: (a) double-arm studies, (b) prospective randomized control trial or retrospective case series, (c) studies included cases among patients with any size (smaller or larger than 400 μm) of MH who had been treated with the inverted ILM flap technique or ILM peeling, (d) anatomical hole closure rate and visual acuity (VA) were reported, and (e) the relevant data was provided. The exclusion criteria included: (a) patients with macular retinoschisis, age-related macular degeneration, retinal detachment, or proliferative diabetic retinopathy, (b) short -term follow-up that is less than three months, (c) nonhuman studies, phase I clinical trials, case reports, editorials, abstracts, reviews, comments and letters, expert opinions, studies without original data and duplicate publications, (d) and studies that lacked a control-treated group for comparison.

### Data extraction

Two investigators (SMMA and SZS) independently extracted the following information from each included study: study characteristics (first author, year of publication, country, sample size and study type) surgical procedure, participant baseline characteristics, minimum diameter of MH, hole closure rate, preoperative and postoperative VA, and follow-up time. Any discrepancies between data extractions were resolved by discussion or consultation with a third investigator (MM).

### Quality assessment

This review included Twenty-six studies: 9 RCTs and 23 retrospective studies. The included RCTs were evaluated for quality using Cochrane Risk of Bias assessment tool [32]. Seven components were assessed: (1) random sequence generation, (2) allocation concealment, (3) blinding of participants and personnel, (4) blinding of outcome assessment, (5) incomplete outcome data, (6) selective reporting, and (7) other bias. According to whether the included studies fully meet the above criteria, we assessed the quality of trials. The methodological quality of each study was assessed based on the Newcastle-Ottawa Scale (NOS) [33], (Range, 0 to 9 stars) for quality of case control studies in meta-analysis. Studies were rated in three areas, including selection, comparability, and exposure. Scores ≥ 5 indicated that the quality of research was relatively high. All items were independently assessed by two investigators (SMMA and SZS), with consensus reached after deliberation or consultation with the third author (SS).

### Statistical analysis

The meta-analysis was carried out based on the guidelines of the preferred reporting items for systematic reviews and meta-analyses (PRISMA) statements [34]. All statistical analyses were performed using R Statistical Software [54] and meta package v4.17-0 [55]. For the rate of MH closure, odds ratios (ORs), and 95% confidence intervals (Cl) were calculated using the Mantel–Haenszel (M-H) method. To compare the evaluation of VA, the mean difference (MD) of preoperative and postoperative measurements between the two surgical treatments were compared using mean difference (MD) and 95% CI.

To assess potential statistical heterogeneity among trials, the Higgins I^2^ statistics were used. The I^2^ statistic reveals the percentage of variation between studies that is due to heterogeneity rather than chance or sampling error. An outcome of over 75% indicates considerable heterogeneity. When the heterogeneity was high, subgroup analysis or sensitivity analysis was used to identify sources of heterogeneity. We used random effect model for the analysis. The results of meta-analysis were visually examined by forest plot, and the potential publication bias was shown by funnel plot and Egger’s test. A *p* < 0.05 was considered statistically significant.

## Results

### Study characteristics

**Fig. 1:**
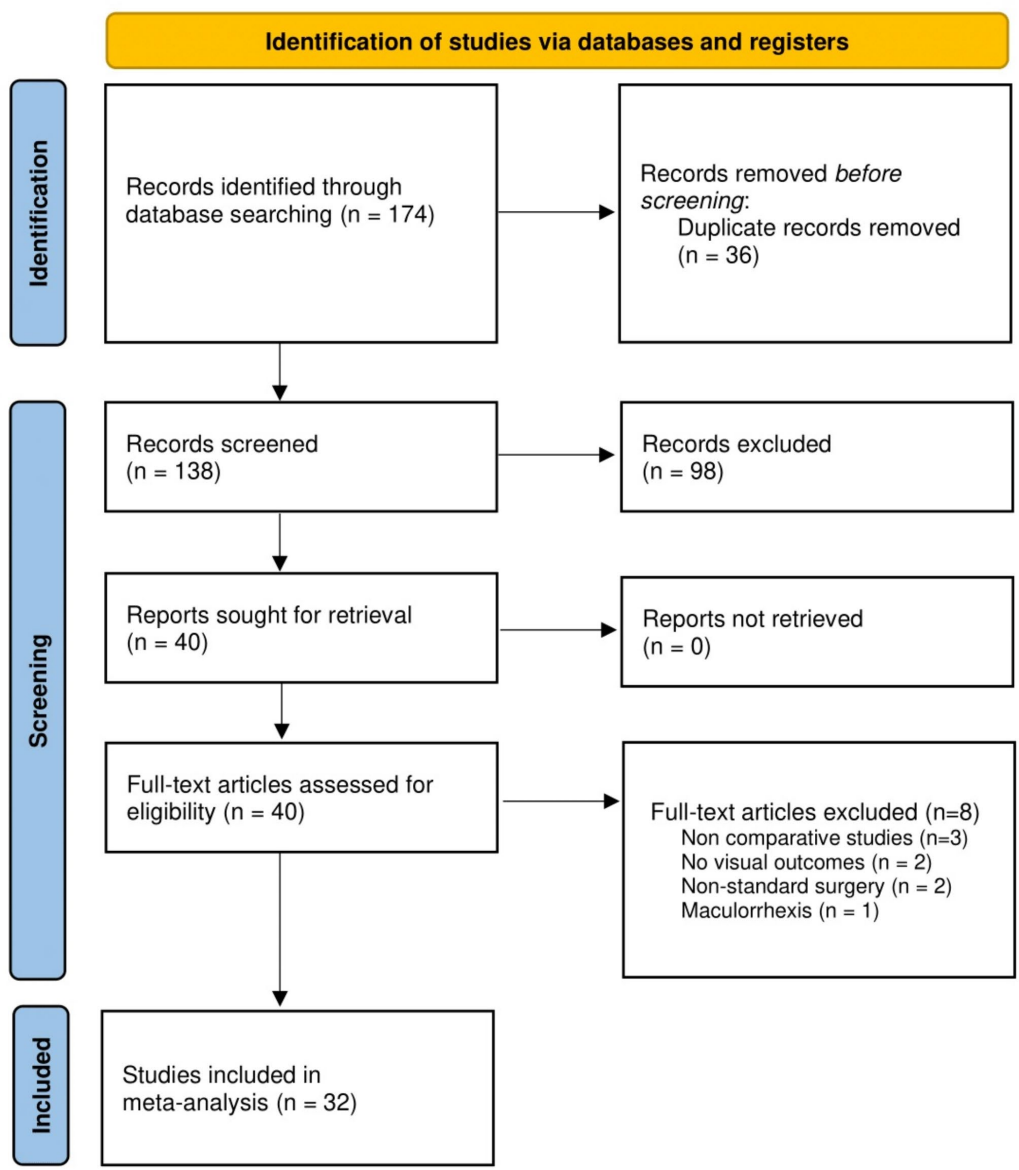
PRISMA Flow Diagram of the Literature Search Process

The detailed flowchart of the study selection process is shown in [Figure. 1]. A total of 174 articles were found after the preliminary literature search, after removing the duplicates the articles were shortlisted first by the topics, then by reading the abstracts, and finally by the way of full text review, leaving a total thirty-two studies [13–30, 35–48] which were included in this meta-analysis.

The main characteristics of the included trials are presented in [Table 1]. Out of the included studies, twenty three were retrospective in nature and nine were RCTs. A total of 2497 eyes were included in this meta-analysis, 1277 in the ILM peeling group and 1220 in the ILM flap group. The mean age ranged from 54.38 to 71 years in the ILM flap group and from 56.26to 72.5 years in the ILM peeling group, the mean minimum diameter of the MH ranged from as low as 269 μm to as high as 803.33 μm in the flap group and 244 to 759.97 μm in the peeling group. The mean follow-up months durations across all studies ranged from 3 to 20.15 months.

**Table 1:** Study characteristics of the included studies

Author	Year	Study location	Study design	Follow-up month	Groups	Eyes, *n*	Mean age, years	Gender (male/female)	Minimum MH diameter (µm)	Hole closure rate, %
Kannan et al. [17]	2018	India	Prospective randomized control trial	6	ILM flap	30	59.37±6.71	11/19	803.33±120.65	90
					ILM peeling	30	61.17±7.42	17/13	759.97±85.01	76.7
Iovino et al. [24]	2018	Italy	Randomized Clinical Trial	6	ILM flap	20	71 ± 8.77 (54–83)	9/11	666.95± 63.7	100
					ILM peeling	20	69 ± 10.4 (49–88)	7/13	664.6± 71.1	100
Manasa et al. [15]	2018	India	Prospective, randomized study	3	ILM flap	43	63.41	20/23	673	95.3
					ILM peeling	48	60.95	22/26	657.5	87.5
Velez−Montoya et al. [23]	2018	Mexico	Prospective randomized controlled trial	3	ILM flap	12	64.2±6.7	NA	608.89±213	91.67
					ILM peeling	12	61.8±9.6	NA	522.22±82.73	91.67
Michalewska et al. [14]	2010	Poland	Prospective, randomized clinical trial	12	ILM flap	50	66	13/33	759	98
					ILM peeling	51	65	8/32	698	88
Ventre et al. [26]	2022	Italy	Randomized Clinical Trial	12	ILM flap	25	62±5	14/11	269 ± 52	100
					ILM peeling	25	64±5	13/12	254 ± 70	100
Agrawal et al. [16]	2022	India	Randomized Clinical Trial	12	ILM flap	75	65.39±4.87	NA	765.6±77.01	100
					ILM peeling	75	64.17±5.95	NA	749.7 ±167.6	93.33
Yamada et al. [30]	2022	Japan	Retrospective, nonrandomized, comparative study	12	ILM flap	21	66.2±10.6	NA	278.6± 80.7	90.5
					ILM peeling	21	66.6 ± 7.0	NA	276.0± 84.5	100
Yilmaz et al. [25]	2021	Turkey	Retrospective, observational, single center study	12	ILM flap	32	68.22 ± 5.38	12/20	494.48 ± 226.5	100
					ILM peeling	25	67.04 ± 7.95	7/18	460.13 ± 160.89	100
Yan et al. [35]	2021	China	Retrospective, non−ran−domized comparative study	6	ILM flap	29	64.28 ± 6.24	13/16	533.41 ± 245.03	100
					ILM peeling	19	67.89 ± 6.19	3/16	502.75 ± 128.59	94.7
Friedrich et al. [47]	2021	German	Retrospective observational study	6	ILM flap	30	65	NA	408.4±157.5	100
					ILM peeling	15	72	NA	287.4±104.9	100
Baumann et al. [21]	2020	United Kingdom	Retrospective, nonrandomized, case series	12	ILM flap	68	NA	NA	560±104	98.53
					ILM peeling	49	NA	NA	504±106	87.76
Bottoni et al. [29]	2020	Italy	Retrospective, nonrandomized comparative study	12	ILM flap	24	69.12±9.75	6/18	652.5±202.85	95.83
					ILM peeling	17	67.58±8.04	6/11	632.11±133.90	100
Ramtohul et al. [13]	2020	France	Retrospective, nonrandomized comparative study	6	ILM flap	23	68.03 ± 9.50	13/10	657.33 ± 172.36	95.65
					ILM peeling	23	65.69 ± 10.25	16/7	574.09 ± 164.68	69.56
Álvarez et al. [44]	2020	Spain	Retrospective case series study	6	ILM flap	12	59.5 (56–67)	6/6	307 (170–453)	91.66
					ILM peeling	16	60 (57–67)	5/11	264 (116–325)	81.25
Lumi et al. [45]	2020	Slovenia	Retrospective study	NA	ILM flap	21	69.8 ± 7.1	6/15	299.2 ± 151.6	95.2
					ILM peeling	17	68.8 ± 5.4	6/9	458.1 ± 130.8	100
Rizzo et al. [43]	2018	Italy	Retrospective, consecutive, nonrandomized comparative study	9.3± 2.03	ILM flap	320	NA	NA	NA	91.3
					ILM peeling	300	NA	NA	NA	78.6
Iturburu et al. [42]	2019	Spain	Retrospective, nonrandomized, comparative study	6	ILM flap	27	NA	NA	NA	92.6
					ILM peeling	39	NA	NA	NA	94.8
Wu et al. [22]	2018	Taiwan	Retrospective study	17.29±20.20	ILM flap	6	62.33±4.18	3/3	297.83±56.55	100
					ILM peeling	8	57.88±12.58	2/6	264.25±87.63	75
Hu et al. [41]	2019	China	Retrospective study	NA	ILM flap	10	58.8±13.8	3/16	NA	100
					ILM peeling	11	59.9±8.5	4/15	NA	81.8
Mete et al. [46]	2017	Italy	Retrospective study	6	ILM flap	34	60.8	NA	NA	94.1
					ILM peeling	36	57.8	NA	NA	61.1
Pak K Y et al. [20]	2017	Korea	Retrospective case series	3	ILM flap	41	65.7±7.5	9/32	590.8±113.1	100
					ILM peeling	51	66.1±6.6	17/34	558.9±106.8	88.2
Yamashita T et al. [19]	2018	Japan	Retrospective case series	6	ILM flap	60	NA	NA	624±108	100
					ILM peeling	105	NA	NA	544±119	92.4
Iwasaki et al. [28]	2018	Japan	Retrospective case series	10.0 ± 4.1	ILM flap	14	65.4±9.0	5/9	655.2±112.1	21.4
					ILM peeling	10	69.9±6.6	2/8	551.1±99.5	70
Narayanan et al. [18]	2018	India and Puerto Rico	Retrospective case series	6	ILM flap	18	60.22±12.09	5/13	577.4±159.4	88.9
					ILM peeling	18	67.50±7.78	8/10	493.8±170.5	77.8
Leisser et al. [27]	2022	Austria	Prospective randomized trial	3	ILM flap	7	71±7	3/4	275±90	100
					ILM peeling	9	67±5	2/7	244±101	100
Ehrhardt et al. [39]	2023	France	Single center, Prospective, open label, randomized controlled clinical trial	3	ILM flap	30	70.5	8/22	405	93.3
					ILM peeling	30	72.5	12/18	375	93.3
Carballéset al. [40]	2023	Spain	Retrospective, consecutive, non−randomized, single−centre study	6	ILM flap	44	70.05 ± 8.14	19/25	387.30 ± 186.81	100
					ILM peeling	36	69.28 ± 9.32	17/19	379.09 ± 191.10	91.67
Ozbek et al. [38]	2023	Turkey	Retrospective study	20.15	ILM flap	18	60.12±11.85	6/12	615.12±255.23	88.9
					ILM peeling	26	62.52±13.54	7/19	454.12±135.10	76.9
Zhang et al. [37]	2023	China	Retrospective study	14.63 ± 14.07	ILM flap	33	54.38±8.41	10/23	600.79±187.85	81.81
					ILM peeling	69	56.26±9.49	28/39	423.55±190.99	86.95
Koçak et al. [48]	2023	Turkey	Retrospective interventional study	6	ILM flap	28	67.5±5.1	11/17	682.0±186.7 (610–882)	96.4
					ILM peeling	32	66.6±5.8	14/18	706.1±232.7 (602–905)	75
Chen et al. [36]	2023	China	Retrospective study	12	ILM flap	15	NA	NA	517.27 ± 175.80	100
					ILM peeling	34	NA	NA	524.27 ± 193.04	100

*Note. ILM* internal limiting membrane, *MH* macular hole, *NA* not available, n number, *µm* micrometer, +- standard deviation, % percentage

### Quality assessment

**Fig. 2:**
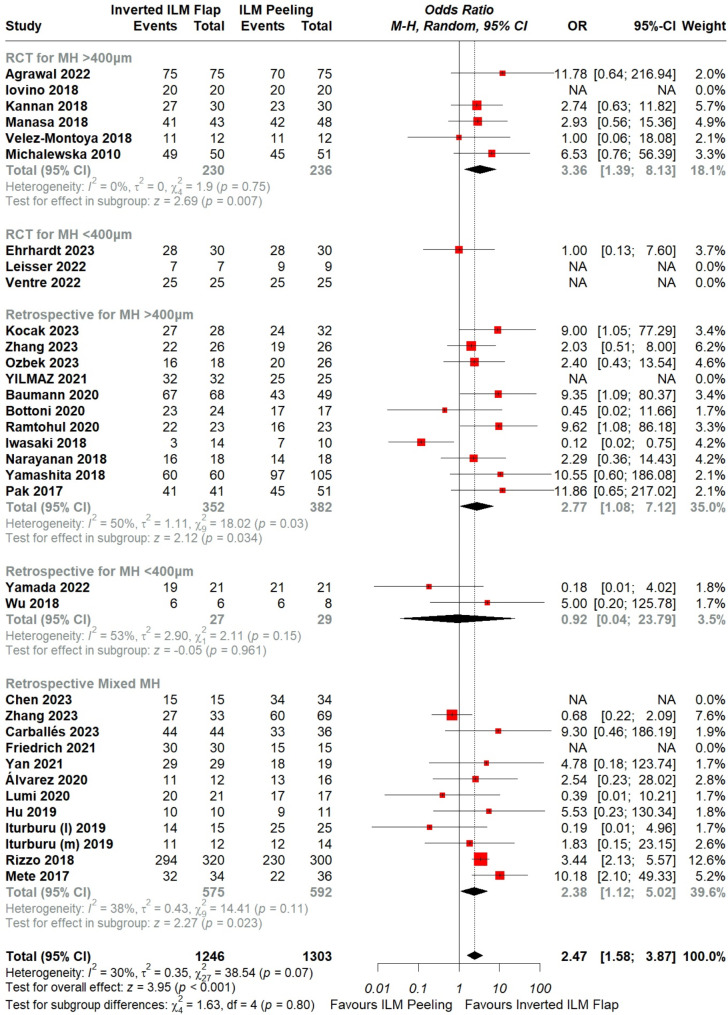
Forest plot of comparison: Inverted ILM flap vs. ILM peeling, outcome: MH closure rate. The subgroup analysis was based on study type I.e., RCT and Retrospective studies with MH sizes of either < 400 μm, > 400 μm and another subgroup based on retrospective studies including mixed sizes

We assessed the quality of the nine RCTs using the Cochrane risk of bias tool and overall, all the studies were found to have low risk of bias and are comprehensively shown in Fig. 2. Six out of nine studies were deemed high quality as they had low risk of bias in all assessing criteria. However, the remaining three studies [14, 24, 39] had unclear risk in different domains.

**Fig. 3:**
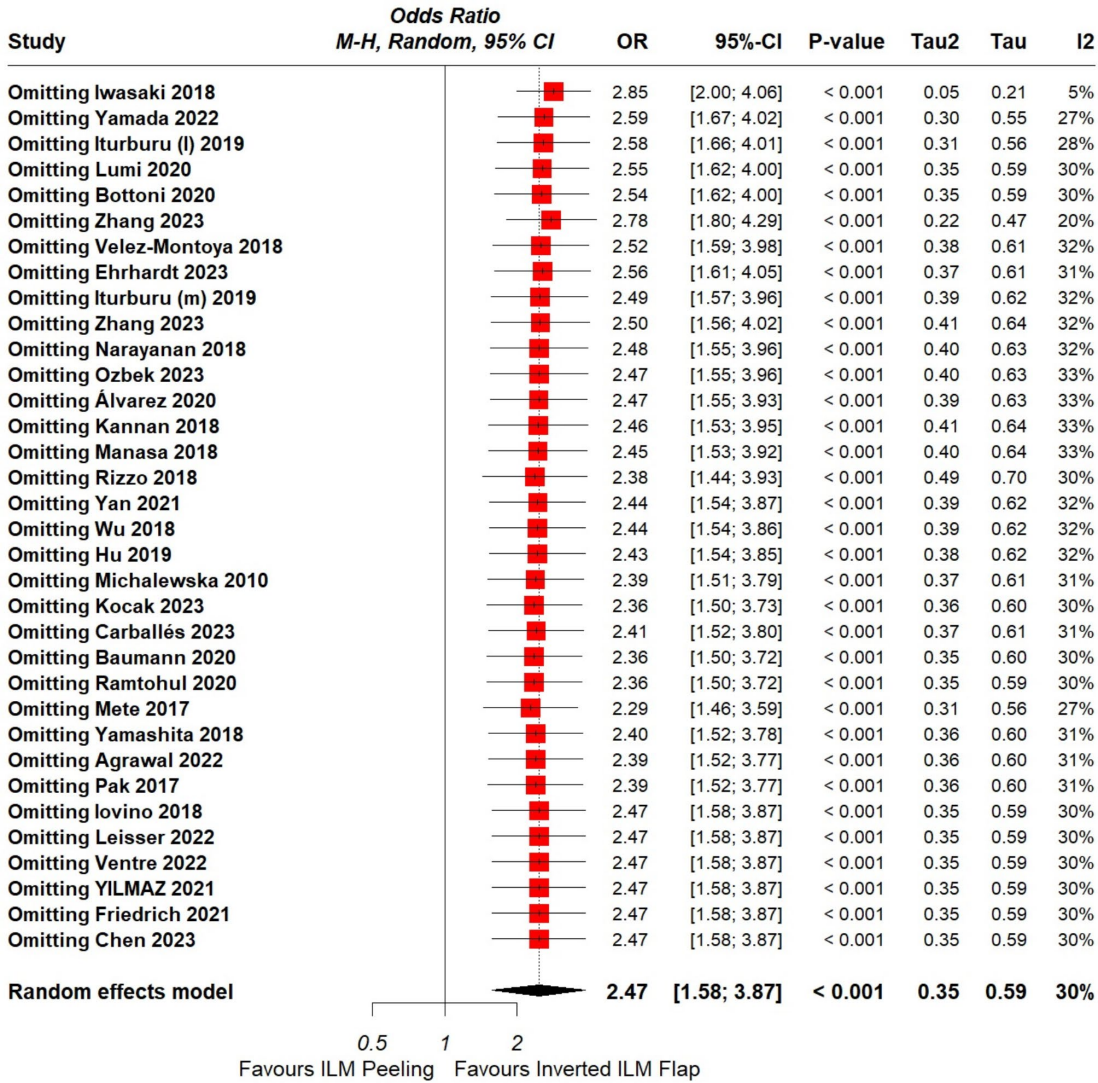
Leave- one-out of MH-closure rate

**Fig. 4:**
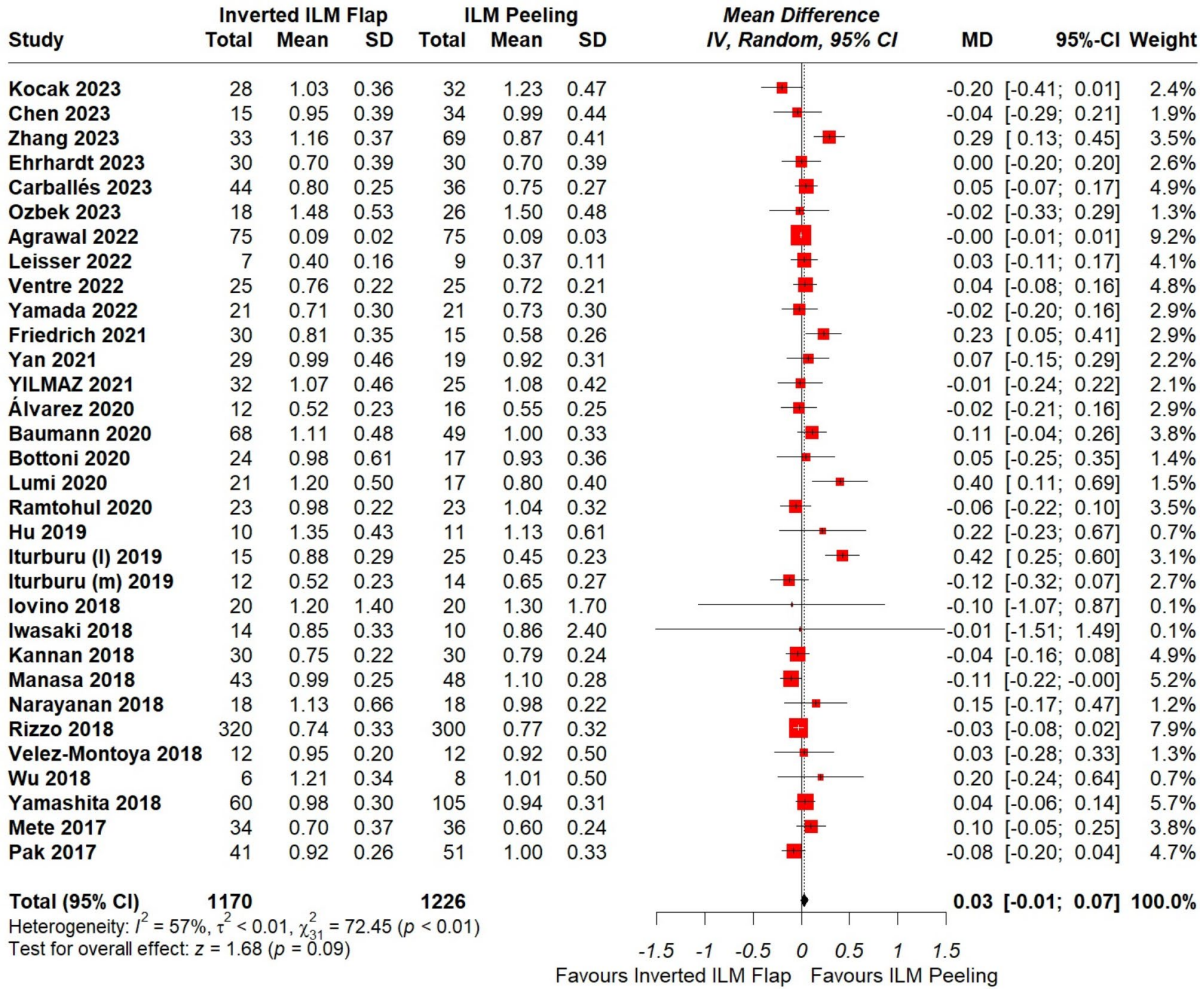
Forest plot of comparison: Inverted ILM flap vs. ILM peeling, outcome: Preoperative visual acuity. Michalewska’s study was not included in the analysis of preoperative VA since SD of preoperative VA was not given

**Fig. 5:**
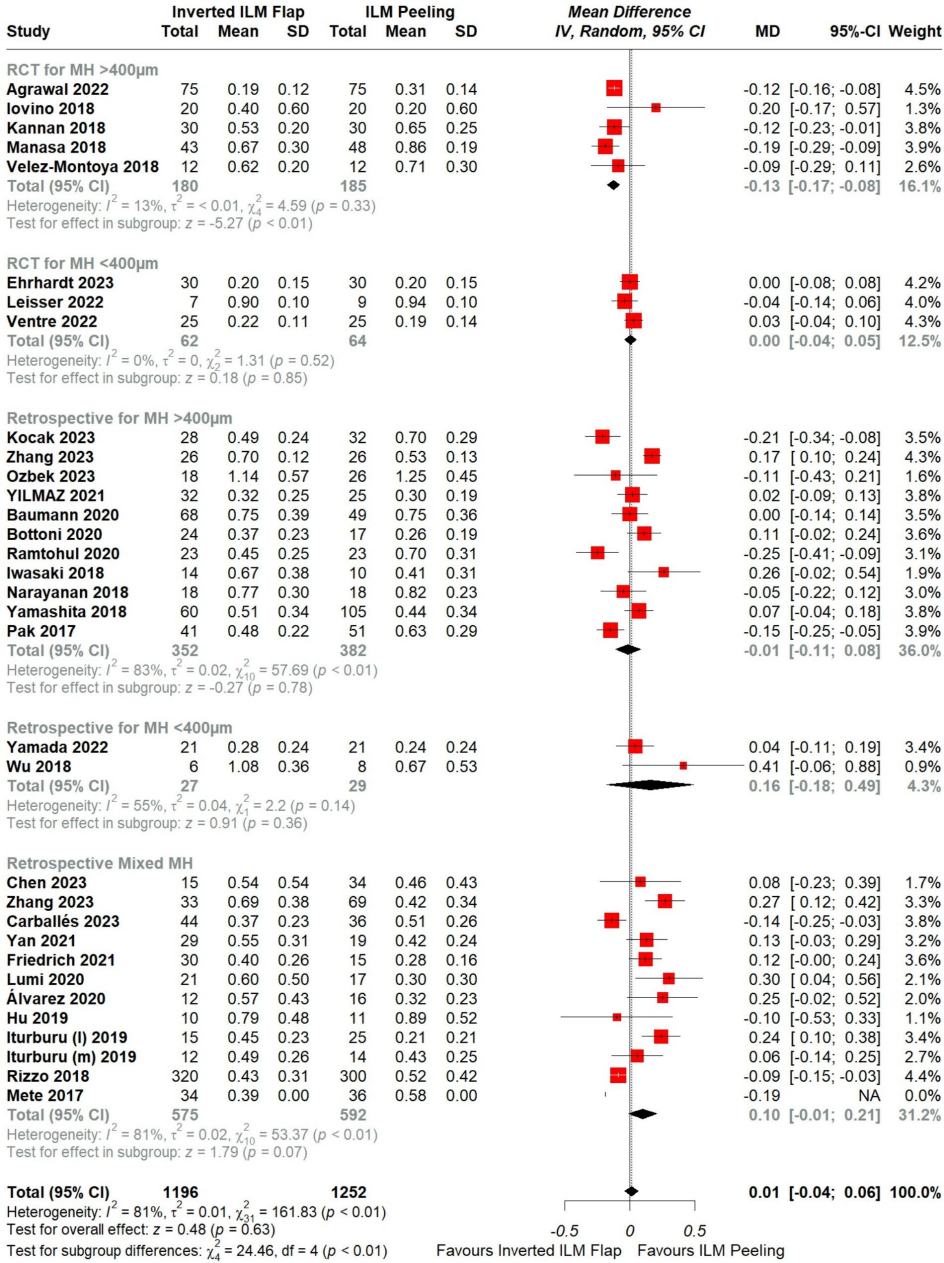
Forest plot of comparison: Inverted ILM flap vs. ILM peeling, outcome: Postoperative visual acuity. The subgroup analysis was based on study type I.e., RCT and Retrospective studies with MH sizes of either < 400 μm, > 400 μm and another subgroup based on retrospective studies including mixed sizes. Michalewska’s study was not included in the analysis of postoperative VA since SD of preoperative VA was not given

**Fig. 6:**
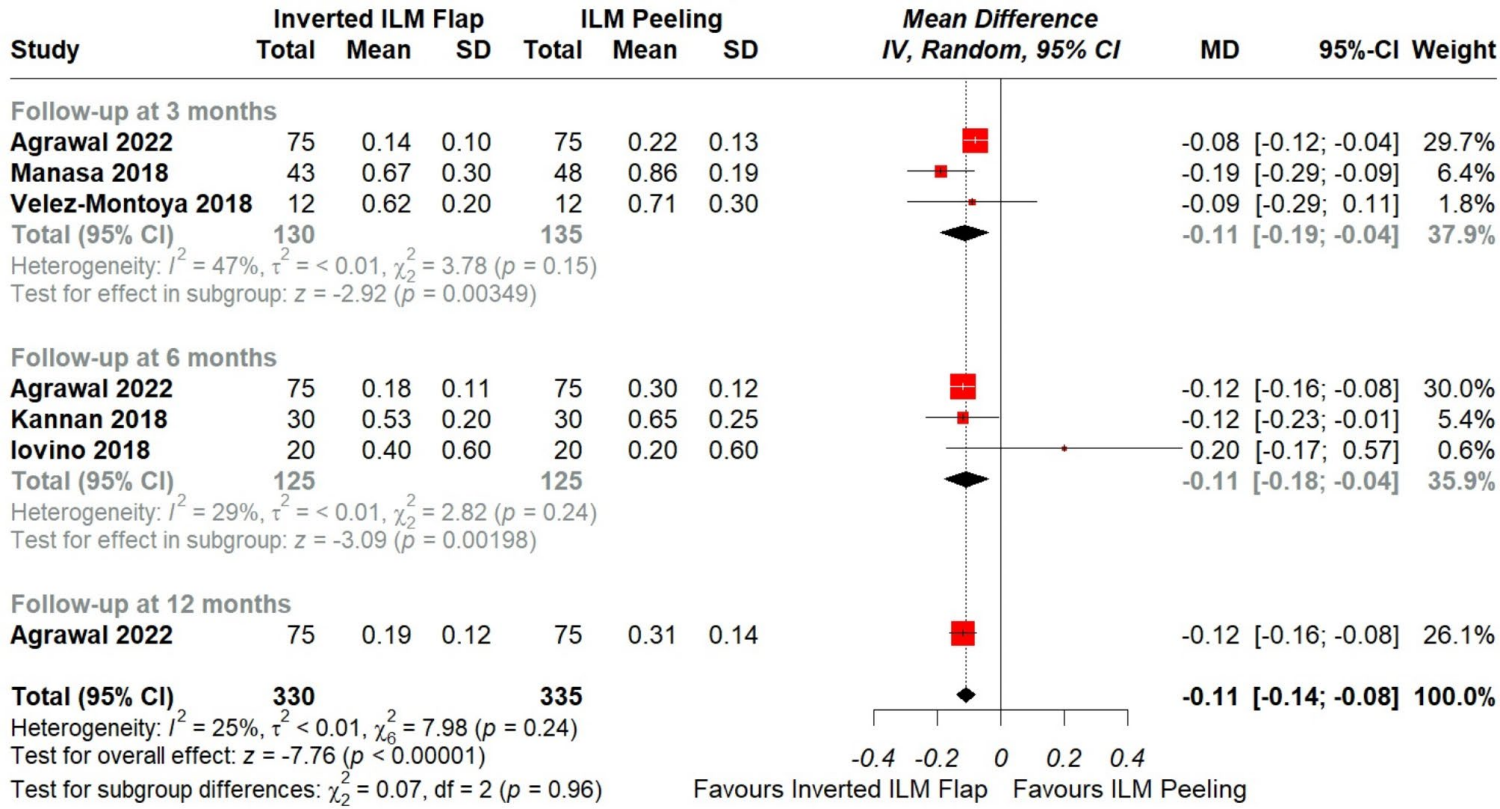
Forest plot of comparison: Inverted ILM flap vs. ILM peeling, outcome: Postoperative VA based on follow-up duration (RCT of > 400 μm). The subgroup analysis was based on follow-up duration including only RCTs with MH > 400 μm that divided the studies into follow-up duration of 3,6 and 12 months. Michalewska’s study was not included in the analysis of postoperative VA since SD of preoperative VA was not given

**Fig. 7:**
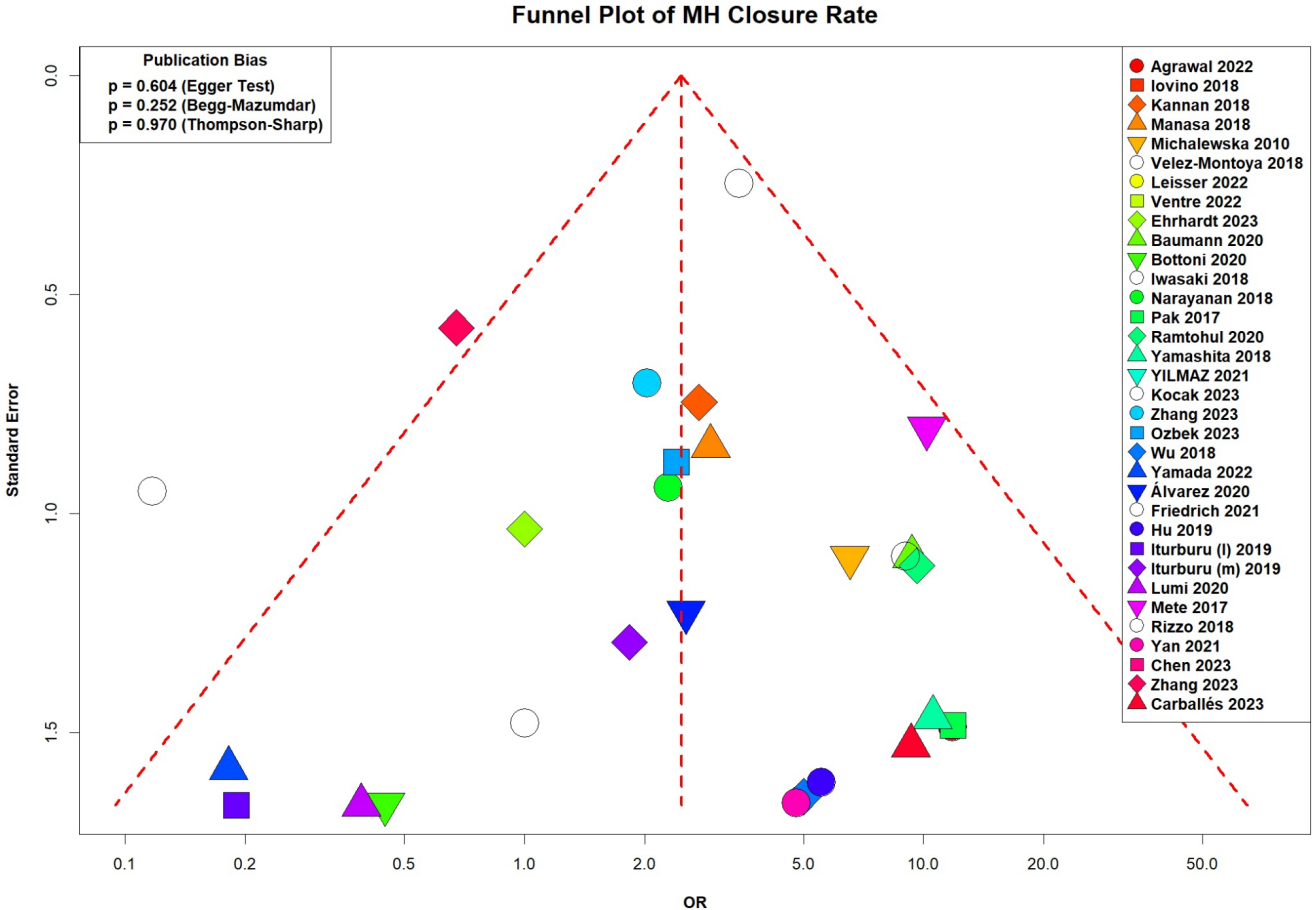
Funnel plot of comparison: Inverted ILM flap vs. ILM peeling, outcome: MH closure rate

As of the the twenty three retrospective studies, all of them were qualitatively assessed using NOS and all studies got five or more stars out of nine except these two studies [28, 30] which secured four out of nine stars as they had concerns regarding the reporting of selection of cases and comparability, shown comprehensively in [Supplementary Table S1].

### Macular hole closure rate

All of the included studies reported this outcome, however, the study by Michalewska et al. [14] did not mention SD for postoperative VA.

The overall pooled data showed significantly higher MH closure rate with inverted ILM flap technique in comparison with the ILM peeling group (OR = 2.47, CI = 1.58 to 3.87; *P* < 0.001; I²= 30%), [Figure. 2]. A leave-one-out analysis was performed, which identified Iwasaki et al. [28] as an outlier. After removing this trial, the overall MH closure rate remained significantly higher with the flap technique (OR = 3.09, CI = 2.30 to 4.15, *P* < 0.001; I^2^ = 5%) and the heterogeneity lowered down to 5%, [Supplementary figure. 2].

Furthermore, the forest plot demonstrated that Rizzo et al. [43] carried significant weightage on the overall result. Leave-one-out analysis was performed, which validated the conclusion in favor of ILM flap technique (OR = 2.38, CI = 1.44 to 3.93, *P* = 0.003; I^2=^ 30%), [Supplementary figure. 3]. A detailed leave-one-out analysis is shown in [Figure. 3].

Subgroup analysis was performed based on the different small (< 400 μm), large (> 400 μm), or mixed MH sizes given in the RCTs and retrospective studies separately. The test for subgroup differences did not reveal a significant difference between the two procedures, (*p* = 0.80), [Figure. 2].

### Preoperative visual acuity

All of the included studies reported the preoperative visual acuity outcome except the study by Michalewska et al. [14] which did not mention standard deviation (SD) of preoperative VA. The forest plot for preoperative visual acuity showed no significant difference between ILM flap and ILM peeling technique (MD = 0.03, 95% CI= -0.01 to 0.07, *P* = 0.09; I^2^ = 57%), [Figure. 4].

### Postoperative visual acuity

All of the included studies reported the postoperative VA outcome after treatment with ILM peeling or inverted ILM flap technique except the study by Michalewska et al. [14] which did not mention SD for postoperative VA.

The meta-analysis of overall pooled data showed postoperative VA with ILM flap technique was statistically insignificant in comparison with the ILM peeling group (MD = 0.01, 95% CI = -0.04 to 0.06, *p* = 0.63; I^2^ = 81%), [Figure. 5]. A detailed leave-one-out analysis was performed; however, it did not explain the high heterogeneity.

Subgroup analysis was performed based on the different small (< 400 μm), large (> 400 μm) or mixed MH sizes given in the RCTs and retrospective studies separately. The test for subgroup differences was statistically significant between ILM flap and ILM peeling groups for postoperative VA, (*p* < 0.01), implicating that the combined variables of study type and MH size significantly impact the pooled results.

In particular, the flap technique was favored over conventional peeling in RCTs with MH sizes > 400 μm, (MD = -0.13, 95% CI = -0.17 to -0.08, *p* < 0.01; I^2^ = 13%), [Figure. 5] in comparison to the other subgroups all of which showed insignificant difference between the two procedures I.e., RCTs with MH sizes < 400 μm (MD = 0.00, 95% CI = -0.04 to 0.05, *p* = 0.85; I^2^ = 0%), [Figure. 5], retrospective studies with > 400 μm (MD = -0.01, 95% CI = -0.11 to 0.08, *p* = 0.78; I^2^ = 83%), [Figure. 5], < 400 μm (MD = 0.16, 95% CI = -0.18 to 0.49, *p* = 0.36; I^2^ = 55%), [Figure. 5] and mixed MH sizes (MD = 0.10, 95% CI = -0.01 to 0.21, *p* = 0.07; I^2^ = 81%), [Figure. 5]. However, there is substantial unexplained heterogeneity between the studies within the retrospective subgroups (> 400 μm: I^2 =^ 83%; <400 μm: I^2^ = 55%; mixed sizes: I^2^ = 81%). Therefore, the validity of the treatment effect estimate for these subgroups is uncertain.

Furthermore, to account for the impact of follow-up duration on the results, we conducted a subgroup analysis that categorized all the RCTs with > 400 μm sizes as they specifically showed significant improvement with the flap technique, based on their follow-up duration. Studies were divided into three categories: follow-up at 3, 6, and 12 months. The overall pooled effect was statistically significant in favor of the flap surgery as compared to peeling (MD = -0.11, 95% CI = -0.14 to -0.08, *p* < 0.01; I^2^ = 25%), [Figure. 6]. A detailed leave-one-out analysis is shown in [Supplementary figure. 5]. The test for subgroup differences for postoperative VA based on follow up duration was not significant between ILM flap and ILM peeling groups (*p* = 0.96).

### Publication bias

The funnel plots for the outcomes of MH closure rate [Figure. 7], and postoperative VA [Supplementary figure. 7] revealed that nearly all of the studies shown by scattered points in the funnel plot were distributed in the middle and top of the baseline and were in the range of the inverted funnel which implies that there is no significant publication bias and the results from this study are reliable. The funnel plot for preoperative VA [Supplementary figure. 6] showed most of the studies clustered at the top of the plot as it is a baseline variable and does not differ much between studies.

Furthermore, an eggers test was performed as the number of articles exceeded ten. Egger’s test for a regression intercept gave a *p*-value of 0.604 for the MH closure rate, indicating no evidence of publication bias.

## Discussion

The objective of this meta-analysis was to assess the efficacy of the relatively new ILM flap technique compared to traditional ILM peeling on anatomic and visual outcomes in patients with any type or size of macular hole without retinal detachment.

Our meta-analysis showed a significant increase in MH closure rate with the ILM flap technique compared to the ILM peeling treated patient population. Additionally, to further evaluate the impact of the study type and macular hole size, subgroup analysis was performed which did not reveal any significant differences with respect to study type and MH size. Moreover, leave-one-out analysis to eliminate the source of heterogeneity was performed removing the study by Iwasaki et al. [28] (as it had a lot of limitations) and this reduced the heterogeneity to a negligible 5%. The study by Rizzo et al. [43] was also removed from the analysis to check the robustness of the overall finding as it carried substantial weightage on the overall result.

ILM flap involves attaching the edges of a hole in the retina to the margins of the ILM. This creates a smooth and uninterrupted scaffold that facilitates gliosis, which in turn allows for the migration and proliferation of glial cells, thereby maintaining the anatomical structure of the foveola. Additionally, the activated Müller cells secrete neurotrophic factors and basic fibroblast growth factors (bFGF), promoting the survival of retinal neurons and facilitating the growth of photoreceptor cells towards the center of the fovea [49]. Furthermore, the ILM flap creates a closed compartment that enables the retinal pigment epithelium to effectively remove sub retinal fluid from the affected area, keeping the hole dry and thus contributing to MH closure [15]. Inverted ILM flap technique may potentially result in a higher rate of anatomical closure compared to the ILM peeling technique, owing to the aforementioned factors.

Regarding the visual outcomes, there was no significant difference in preoperative VA between the two groups, which negated any pre-intervention effects on postoperative VA. While for the postoperative VA, the forest plot did not indicate any significant benefit of the flap technique over the other technique overall.

However, subgroup analysis based on study type and MH size showed that eyes treated with the ILM flap technique in RCTs with MH size > 400 μm exhibited significantly better postoperative VA than those treated with ILM peeling compared to all the other subgroups, suggesting the superiority of the use of ILM flap technique for larger MH repair. However, further research is warranted to fully establish the potential benefits of this technique over the conventional peeling procedure. This may be explained by some previous studies demonstrating the inadequacy of the conventional peeling in resulting in a type 1 closure and has a higher rate of a V or a W shaped closure which is associated with degradation of the photoreceptor layer, damage to the retinal pigment epithelium, and depletion of foveal tissue, as compared to the flap technique for large MHs [50].

We further sought to explore the differences in postoperative VA between the two surgical techniques based on follow-up duration set in RCTs with MH sizes > 400 μm I.e., postoperative VA at 3, 6, and 12 months.

ILM flap was again found to be significantly better than ILM peeling based on follow up duration. The reason for this could be the higher MH closure rate in the initial months with the flap technique and the observed proliferative processes associated with MH healing, such as Muller cell proliferation, regeneration of retinal tissue, photoreceptor migration, and repositioning [23, 51–53]. However, it should be mentioned that the included studies did not have a long follow up duration except one study by Agrawal et al. [16] which followed the treated participants till 12 months. Hence, more studies with longer follow-up periods are needed to further confirm this finding.

Finally, this meta-analysis stands out as a comprehensive study, using R core [54] encompassing a total of nine RCTs and twenty three retrospective studies, with a wide range of MH types and sizes. In contrast to previous studies, we did not limit our analysis to a specific MH size or type including idiopathic as well as myopic MH. This approach allowed us to gain a broader understanding and adds additional merit to our study. Moreover, we performed subgroup analysis based on the type of study and the size of the MH, which has not been previously conducted. While performing literature review, we found three similar meta-analysis on this topic, all of them focused on randomized clinical trials. However, our study included both randomized trials and retrospective studies. A comparison of previously published meta-analysis is presented in Table 2 [55–57]. Ghoraba et al. [56] did a sensitivity analysis better to separate type II closure from type I as they found type I closure is generally more predictive of good visual recovery.

**Table 2:** Comparison of key findings in previously published meta-analyses

Feature	Ghoraba et al. (2023)	Chen et al. (2020)	Yu et al. (2021)
Type of Study	Systematic Review and meta-analysis of RCTs	Systematic Review Meta-analysis of RCTs	Systematic Review Meta-analysis of RCTs
Author’s affiliations	USA and Egypt	USA and China	China
Number of Studies Included	4 RCTs	4 RCTs	5 RCTs
Sample size	135 vs. 141	135 vs. 141	155 vs. 161
Macular Hole Closure Rate	**Higher** with ILM flap	**Higher** with ILM flap	**Higher** with ILM flap
Visual Acuity Improvement	**Better** with ILM flap	**Better** with ILM flap	**Better** with ILM flap
Complication Rate	Similar between techniques	Similar between techniques	Similar between techniques
Conclusion	ILM flap has better anatomic and visual outcomes	ILM flap improves closure and function	ILM flap is superior for large macular holes
Sensitivity analyses	Presented for type 1 vs. type 2 closure	Not presented	Not presented

### Limitations

There were some limitations with this study that must be considered when referring to the outcomes of this meta-analysis. Firstly, in this meta-analysis retrospective studies were included alongside RCTs, which may have inherent possibilities of selection bias. Secondly, the comparison of primary and secondary endpoints revealed some heterogeneity among the studies, although we conducted sensitivity analysis and subgroup analysis to alleviate the heterogeneity, we were not able to eliminate it. Thirdly, the follow-up periods in the trials that were included were inadequate for making improved observations of VA recovery in the long term. Fourthly, the type of closure (i.e., type 1 and 2) may potentially influence the postoperative visual acuity outcome. However, we were unable to perform a subgroup analysis for these variables due to insufficient data. Lastly, factors such as the extent of ILM peeling, toxicity of the ILM stain, type of dye used for ILM staining, duration of air or longer-acting gas tamponade, postoperative face-down timing, and the specific form of ILM flap technique across studies could potentially serve as sources of heterogeneity. Unfortunately, due to the scarcity of data on these factors, we were unable to carry out a subgroup analysis. Hence, more original studies with large sample sizes and longer follow-up measurement for improved VA to evaluate these variables in detail are required. The manuscript was available as a pre-print publication as well [58].

## Conclusion

Our meta-analysis comparing ILM flap technique and ILM peeling for the treatment of any type or size of MH showed that the ILM flap technique has a higher closure rate than ILM peeling. Notably, the improvement in VA with the flap technique was significant compared to ILM peeling specifically for larger MHs and based on follow up duration. Our findings support the use of the ILM flap technique for all sizes of MHs, and especially the larger MHs, but more large-scale and longer duration studies are needed to validate the efficacy of these findings especially running a multicenter trial with standardized surgical technique and large numbers focused on small and medium full thickness macular hole (FTMH) may be more novel.

## Electronic supplementary material

Below is the link to the electronic supplementary material.

**Supplementary Material 1**: **Supplementary Fig.1**. Risk of bias summary. The Cochrane “risk of bias” tool was used for quality assessment.

**Supplementary Material 2**: **Supplementary Fig.2**. Forest plot of comparison between inverted ILM flap vs. ILM peeling, outcome: MH closure rate (excluding Iwasaki). Sensitivity analysis showed Iwasaki’s study to be the cause of heterogeneity.

**Supplementary Material 3**: **Supplementary Fig.3**. Forest plot of comparison: Inverted ILM flap vs. ILM peeling, outcome: MH closure rate (excluding Rizzo). Leave one out analysis was performed excluding Rizzo’s study confirming the robustness of our study.

**Supplementary Material 4**: **Supplementary Fig.4**. Leave- one-out of Postoperative visual acuity.

**Supplementary Material 5**: **Supplementary Fig.5**. Leave- one-out of Postoperative visual acuity for RCT > 400μm (excluding Iovino).

**Supplementary Material 6**: **Supplementary Fig.6**. Funnel plot of comparison: Inverted ILM flap vs. ILM peeling, outcome: Preoperative visual acuity.

**Supplementary Material 7**: **Supplementary Fig.7**. Funnel plot of comparison: Inverted ILM flap vs. ILM peeling, outcome: Postoperative visual acuity.

**Supplementary Material 8**: **Supplementary Table S1**. New Castle Ottawa Scale For Assessment Of Publication Bias Of Non-RCT Studies.

## Data Availability

There are no new datasets generated in this article. All data supporting the conclusions of this article is included in the supplementary material.

## Abbreviations

BCVA: Best Corrected Visual Acuity
FTMH: Full thickness macular hole
bFGF: Basic fibroblast growth factors
MH: Macular hole
ILM: Internal limiting membrane
logMAR: Logarithm of minimal angle of resolution
MD: Mean difference
NOS: Newcastle-Ottawa Scale
OR: Odds ratio
RCT: Randomized control trial
SD: Standard deviation
VA: Visual acuity
RD: Retinal Detachment
