# Supplementary figures and images for: Critical analysis of macular hole repair techniques: a comprehensive systematic review and meta-analysis comparing internal limiting membrane flap and internal limiting membrane peeling for any size of macular hole
Source: BMC Ophthalmol. 2025 Apr 7;25:174. doi: 10.1186/s12886-025-04011-0 (PMC11974160; doi:10.1186/s12886-025-04011-0)

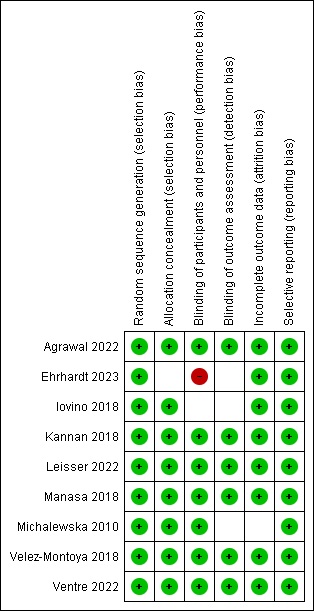

Supplement: Supplementary file 1 — Supplementary Material 1: Supplementary Fig.1. Risk of bias summary. The Cochrane “risk of bias” tool was used for quality assessment. [file 12886_2025_4011_MOESM1_ESM.jpg]

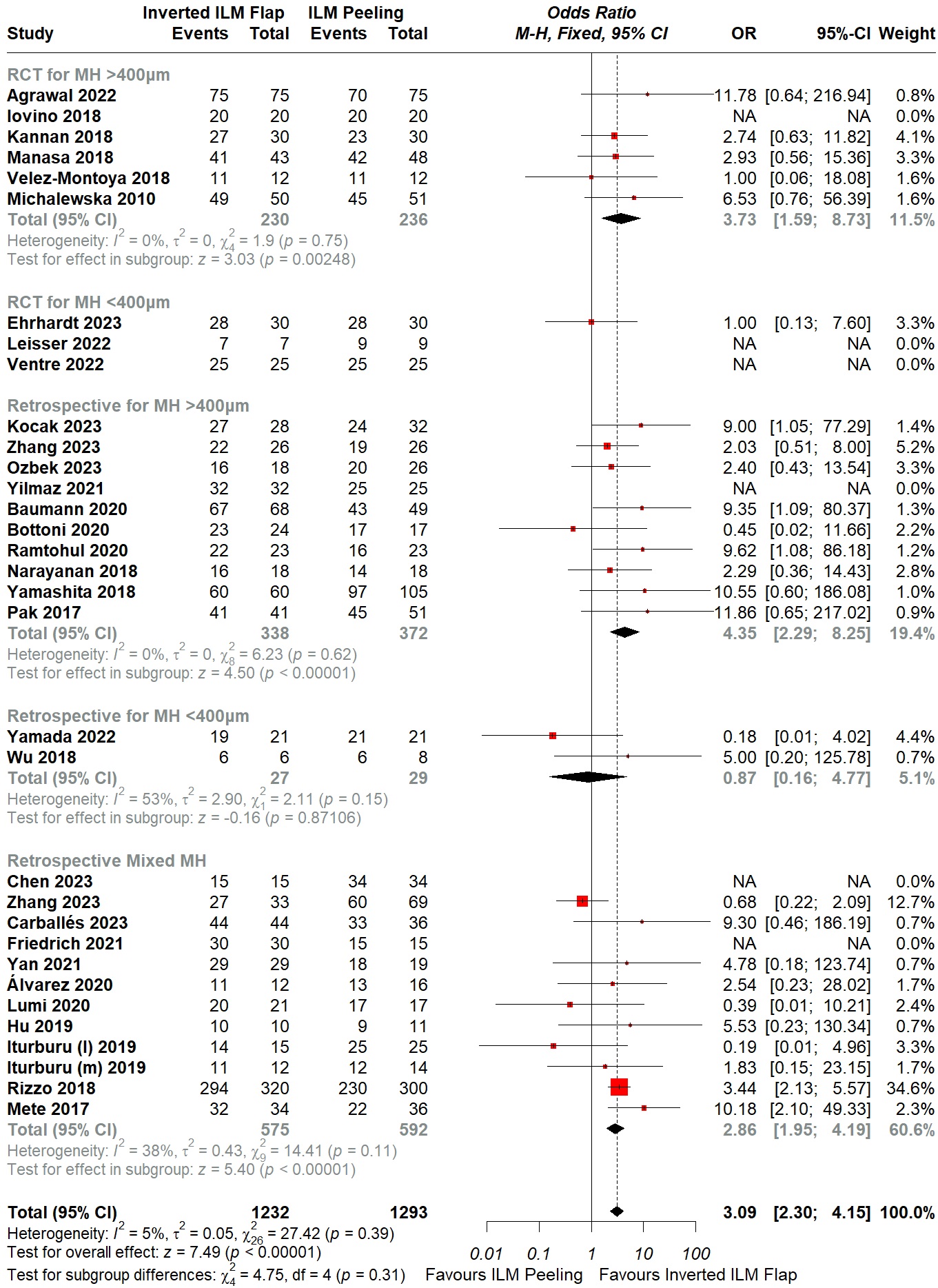

Supplement: Supplementary file 2 — Supplementary Material 2: Supplementary Fig.2. Forest plot of comparison between inverted ILM flap vs. ILM peeling, outcome: MH closure rate (excluding Iwasaki). Sensitivity analysis showed Iwasaki’s study to be the cause of heterogeneity. [file 12886_2025_4011_MOESM2_ESM.jpg]

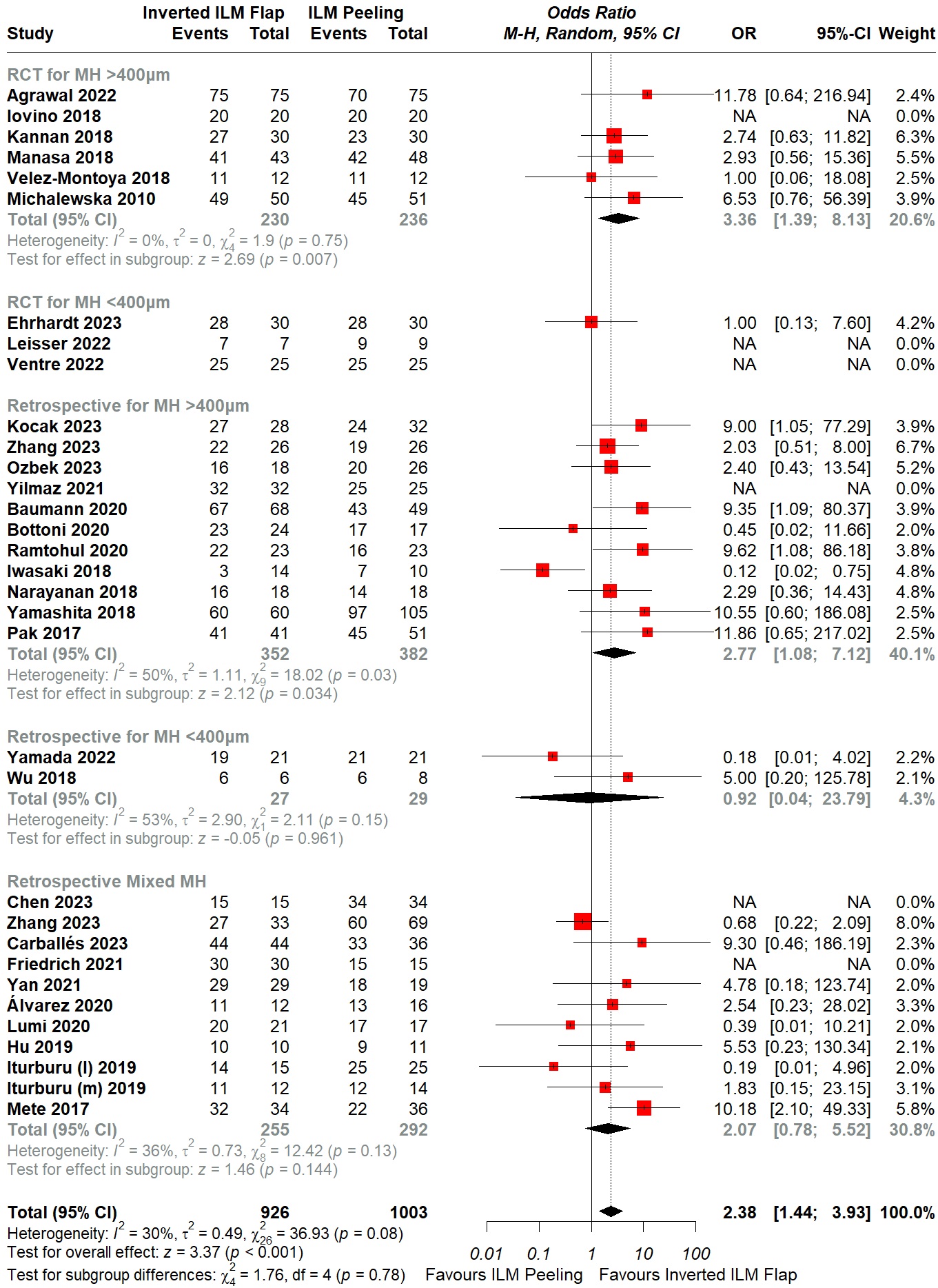

Supplement: Supplementary file 3 — Supplementary Material 3: Supplementary Fig.3. Forest plot of comparison: Inverted ILM flap vs. ILM peeling, outcome: MH closure rate (excluding Rizzo). Leave one out analysis was performed excluding Rizzo’s study confirming the robustness of our study. [file 12886_2025_4011_MOESM3_ESM.jpg]

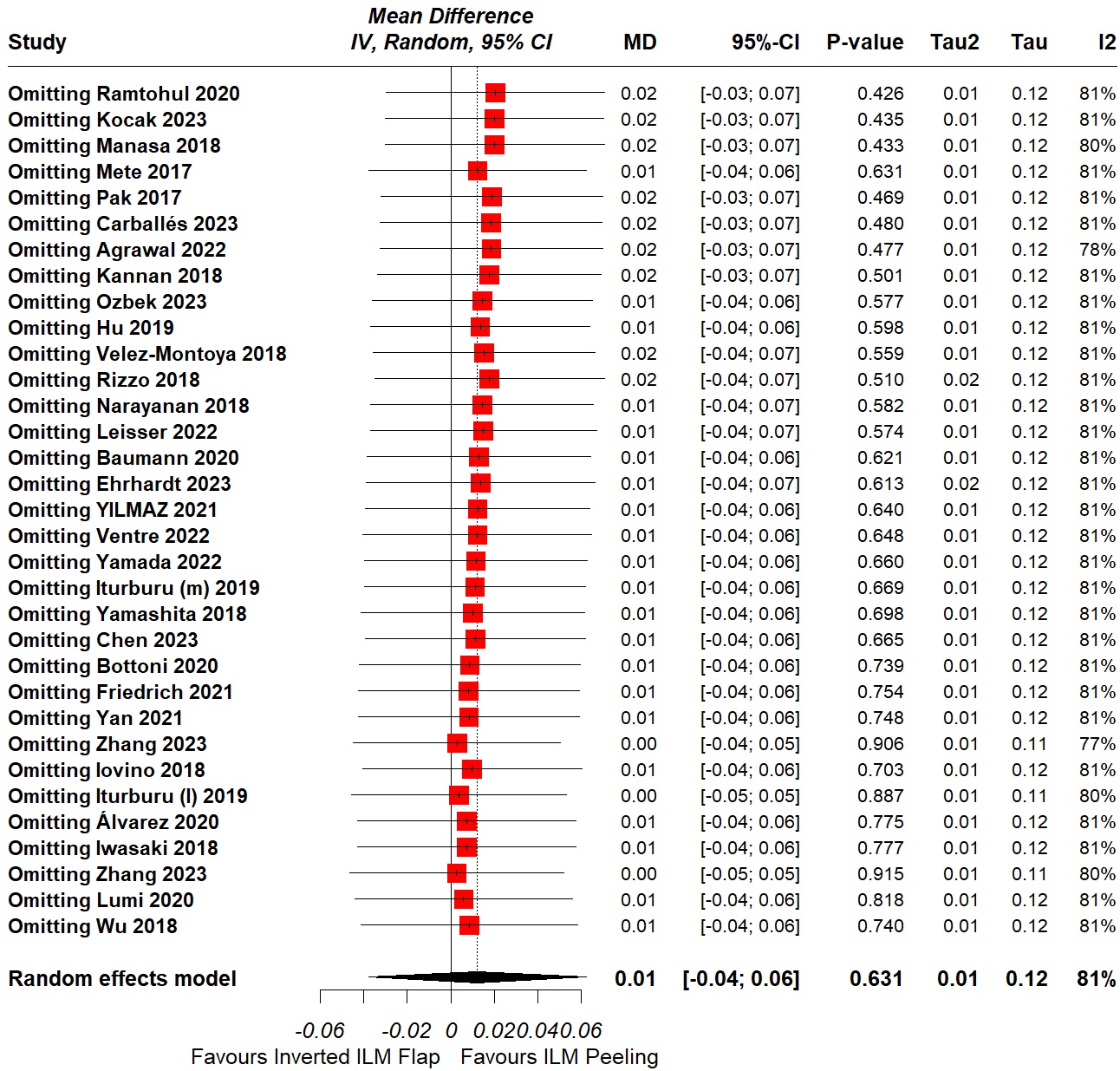

Supplement: Supplementary file 4 — Supplementary Material 4: Supplementary Fig.4. Leave- one-out of Postoperative visual acuity. [file 12886_2025_4011_MOESM4_ESM.jpg]

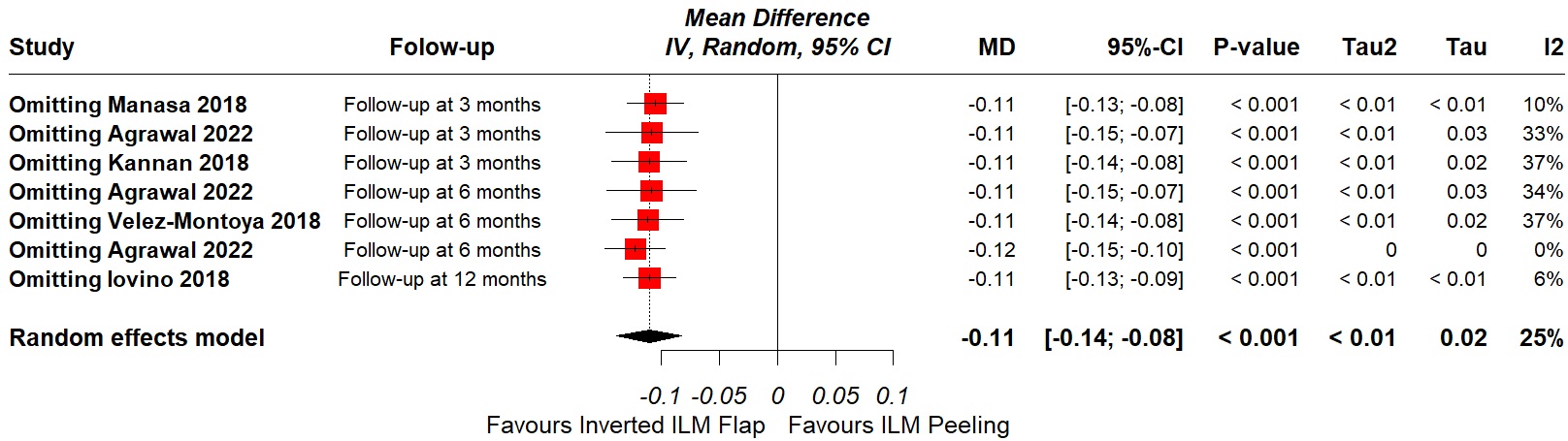

Supplement: Supplementary file 5 — Supplementary Material 5: Supplementary Fig.5. Leave- one-out of Postoperative visual acuity for RCT > 400μm (excluding Iovino). [file 12886_2025_4011_MOESM5_ESM.jpg]

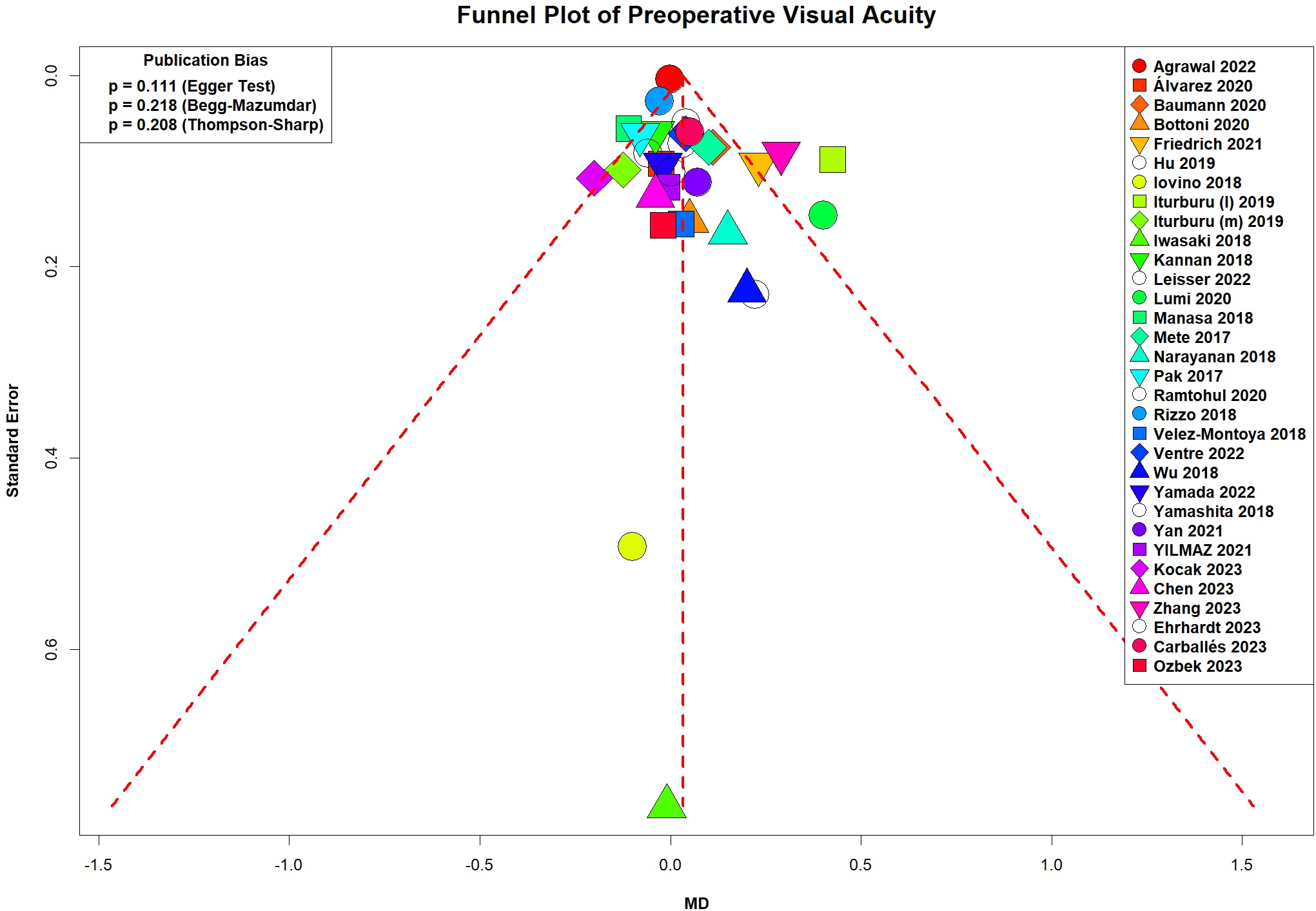

Supplement: Supplementary file 6 — Supplementary Material 6: Supplementary Fig.6. Funnel plot of comparison: Inverted ILM flap vs. ILM peeling, outcome: Preoperative visual acuity. [file 12886_2025_4011_MOESM6_ESM.jpg]

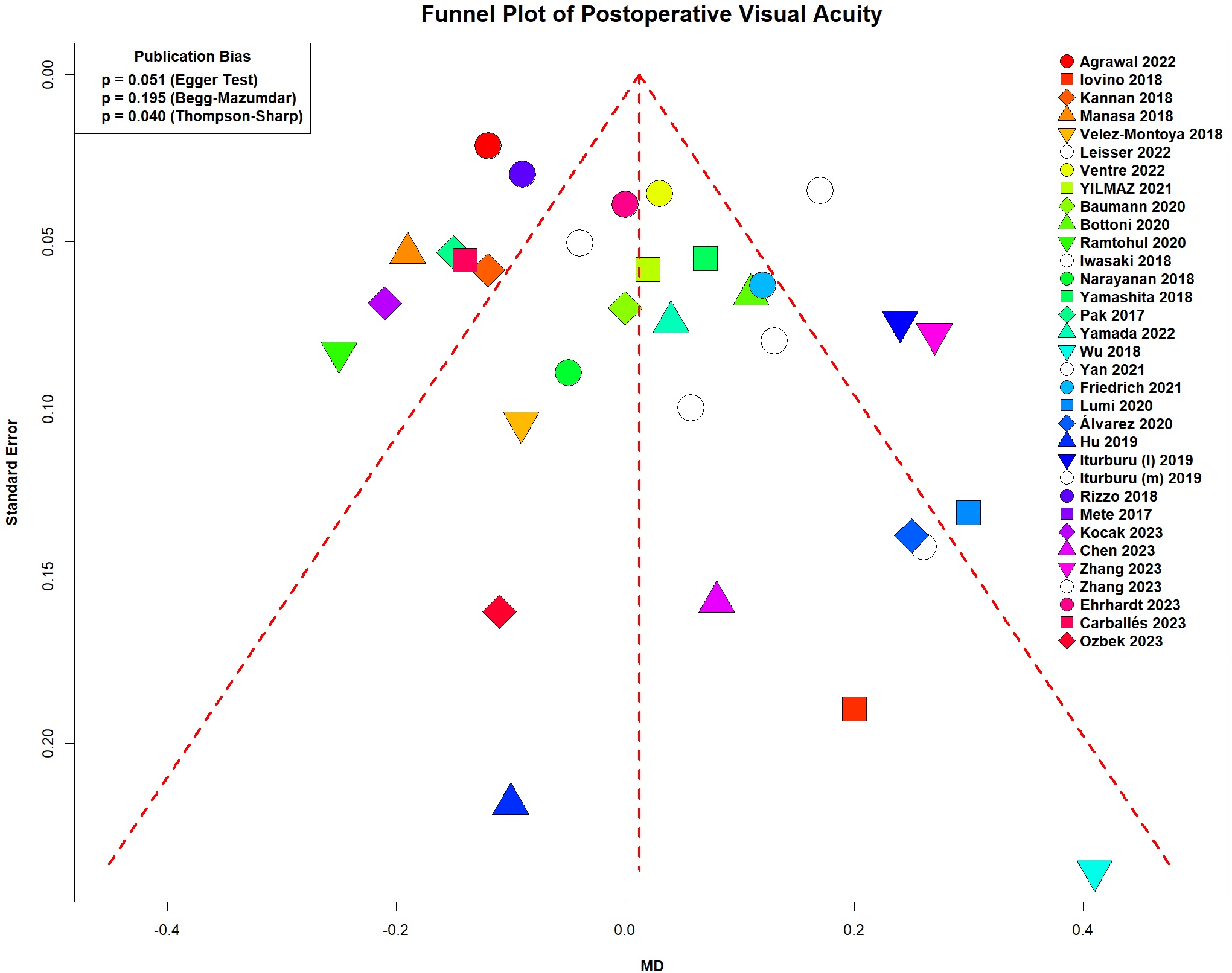

Supplement: Supplementary file 7 — Supplementary Material 7: Supplementary Fig.7. Funnel plot of comparison: Inverted ILM flap vs. ILM peeling, outcome: Postoperative visual acuity. [file 12886_2025_4011_MOESM7_ESM.jpg]
