# Supplementary material for: Critical analysis of macular hole repair techniques: a comprehensive systematic review and meta-analysis comparing internal limiting membrane flap and internal limiting membrane peeling for any size of macular hole
Source: BMC Ophthalmol. 2025 Apr 7;25:174. doi: 10.1186/s12886-025-04011-0 (PMC11974160; doi:10.1186/s12886-025-04011-0)
Supplement: Supplementary file 8 — Supplementary Material 8: Supplementary Table S1. New Castle Ottawa Scale For Assessment Of Publication Bias Of Non-RCT Studies. [file 12886_2025_4011_MOESM8_ESM.docx]

**Supplementary Table S1**

*New Castle Ottawa Scale For Assessment Of Publication Bias Of Non-RCT Studies*

| Study | Selection | Comparability | Exposure | Total |
| --- | --- | --- | --- | --- |
| Carballés 2023 [40] | 3 | 1 | 2 | 6 |
| Ozbek 2023 [38] | 4 | 1 | 2 | 7 |
| Chen 2023 [36] | 4 | 1 | 3 | 8 |
| Koçak 2023 [48] | 3 | 1 | 2 | 6 |
| Zhang 2023 [37] | 4 | 1 | 2 | 7 |
| Yamada 2022 [30] | 3 | 2 | 2 | 6 |
| Yilmaz 2021 [25] | 2 | 2 | 3 | 7 |
| Yan 2021 [35] | 2 | 2 | 2 | 6 |
| Friedrich 2021 [47] | 2 | 2 | 3 | 7 |
| Alvarez 2020 [44] | 3 | 2 | 2 | 7 |
| Bauman 2020 [21] | 2 | 2 | 2 | 6 |
| Bottoni 2020 [29] | 2 | 2 | 2 | 6 |
| Ramtohul 2020 [13] | 2 | 2 | 2 | 6 |
| Lumi 2020 [45] | 3 | 2 | 2 | 7 |
| Iturburu 2019 [42] | 3 | 2 | 2 | 7 |
| Hu 2019 [41] | 2 | 2 | 2 | 6 |
| Yamashita 2018 [19] | 2 | 2 | 1 | 5 |
| Wu 2018 [22] | 2 | 2 | 2 | 6 |
| Iwasaki 2018 [28] | 2 | 1 | 1 | 4 |
| Narayanan 2018 [18] | 2 | 2 | 2 | 6 |
| Rizzo 2018 [43] | 1 | 1 | 2 | 4 |
| Pak 2017 [20] | 2 | 2 | 2 | 6 |
| Mete 2017 [46] | 2 | 2 | 2 | 6 |
